# The impact of COVID-19 quarantine on dietary habits and physical activity in Saudi Arabia: a cross-sectional study

**DOI:** 10.1186/s12889-021-11540-y

**Published:** 2021-07-30

**Authors:** Manar Abduljalil Bakhsh, Jomana Khawandanah, Rouba Khalil Naaman, Shoug Alashmali

**Affiliations:** 1 Clinical Nutrition Department, Faculty of Applied Medical Sciences, King Abdulaziz University, P.O. Box 80215, Jeddah, 21589 Saudi Arabia; 2 Section for Nutrition Research, Department of Metabolism, Digestion and Reproduction, Faculty of Medicine, Imperial College London, London, UK

**Keywords:** COVID-19, Dietary habits, Nutrition, Physical activity, Quarantine, Weight change

## Abstract

**Background:**

The COVID-19 pandemic has forced governments around the world to impose strict hygiene and national lockdown measures, which in turn has changed the dietary and lifestyle habits of the world’s population. Thus, the aim of this study is to evaluate whether dietary and physical activity behaviors of Saudi Arabia’s adult population changed during the COVID-19 quarantine.

**Methods:**

An electronic questionnaire which assessed changes in body weight, dietary habits, and physical activity of Saudi Arabia’s adult population (*n* = 2255) during the COVID-19 quarantine was distributed on social media between June and July 2020. To test the differences between changes in dietary and physical activity behaviors in relation to changes in body weight a Chi-square test was used.

**Results:**

Over 40 and 45% of participants reported eating and snacking more, respectively, which led to weight gain in around 28%. Most participants reported that they consumed home-cooked (73%) and healthy meals (47%), while only 7% reported that they consumed foods from restaurants. Feelings of boredom and emptiness (44%) and the availability of time for preparing meals (40%) were the main reasons for changing dietary habits. Honey (43%) and vitamin C (50%) were the most consumed immune-boosting food and dietary supplement, respectively. COVID-19 also had a negative impact on physical activity, lowering the practice in 52% subjects, which was associated with significant weight gain (*p* < 0.001).

**Conclusion:**

Assessing the changes to the population’s dietary habits and physical activity during the lockdown will help predict the outcome of the population’s future health and wellbeing after the pandemic.

**Supplementary Information:**

The online version contains supplementary material available at 10.1186/s12889-021-11540-y.

## Background

The coronavirus disease, also known as COVID-19, is a serious acute respiratory syndrome, which has resulted in a spread of moderate to severe illness globally [1]. In March 2020, the World Health Organization (WHO) declared COVID-19 a global pandemic and public health threat [2]. In Saudi Arabia, the number of COVID-19 cases has been increasing since February 2020. To eradicate the spread of the virus, the Saudi Arabian government has established specific lockdown precautions policies in March 2020: forcing individuals to quarantine themselves at home, suspending travel, imposing social distancing rules, and banning attendance at workplaces, schools, restaurants, shops, and gyms [3, 4]. During quarantine, changes in health and socioeconomic status have been reported worldwide [5]. Other negative consequences related to the stay-at-home restrictions include anxiety, stress, depression, emotional eating (as well as other dietary changes), and limited physical activity in the adult population [6]. These consequences were accompanied with higher food intake, sleeping disorders, and weight changes [7].

Nutritional status during the COVID-19 quarantine has been severely altered worldwide and become a priority in this stressful situation. Nutritional deficiencies of macronutrients and micronutrients are linked with suppressed immunity and increased infection susceptibility. Consuming supportive nutrients and antioxidants, such as zinc, iron, and vitamins A, D, E, and C is essential for maintaining healthy immune system [8, 9]. Although negative dietary habits during quarantine have been reported in several countries, such as Poland and Italy [10, 11], COVID-19 confinement has also led to healthy dietary behaviors in Spain, as reflected by high compliance to the Mediterranean diet. This improvement could play a positive role in preventing chronic diseases and COVID-19 related complications if persistent [12]. However, information related to the impact of the COVID-19 quarantine on dietary habits in Saudi Arabia is lacking.

In regards to physical activity, the WHO currently recommends that adults not diagnosed with COVID-19 or experiencing respiratory symptoms should perform at least 150 min of moderately intense or 75 min of vigorously intense physical activity weekly [13]. Despite these recommendations, a general decline in the level of physical activity was found in studies assessing populations’ physical activity during the COVID-19 quarantine particularly in Spain and Poland [10, 12]. Information on how COVID-19 related quarantine affects physical activity level of population in Saudi Arabia is unidentified.

Limited information is available on the effect quarantine has had on perceived weight changes, dietary patterns and physical activity before and during the pandemic period. This present study will provide a better understanding of how diet and health in Saudi Arabia has been affected compared to other countries, considering the importance that dietitians can play in such epidemics, and set suitable recommendations for the future. The study’s aim is to assess changes of body weight, dietary intake habits, and physical activity of Saudi Arabia’s adult population during the COVID-19 quarantine. In line with previous studies [6, 10, 14], we hypothesized that the majority of the study participants would report changes in their weight and most likely weight gain, and a negative effect of the COVID-19 quarantine on participants’ dietary habits and physical activity levels.

## Methods

### Study design

A cross-sectional study was approved by the Unit of the Biomedical Ethics Research Committee at King Abdulaziz University (Jeddah, Saudi Arabia) (reference no. 355–20). An informed consent was provided by all study participants at the beginning of the online questionnaire.

### Sample size calculation

The Epi Info online sample size calculator [15] was used to compute the required sample size based on both a previous study conducted in Saudi Arabia [16] and the Saudi General Authority for Statistics in 2019 [17]. The anticipated dropout rate was a 20%, with a 99.99% confidence level, a 5% margin error, and a design effect of 1; therefore, 1817 participants were required.

### Participants and recruitment

The inclusion criteria were citizens and residents of Saudi Arabia age ≥ 18 years old, either male or female. Participants were asked to fill out an anonymous electronic questionnaire that was created via Google Forms and distributed on various platforms, such as WhatsApp, Twitter, and email. In order to reach out to all society members, the questionnaire link was sent to the authors’ relatives, friends, and neighbors to participate in the study and to share the link with their contacts.

### The questionnaire

An online questionnaire was designed to assess and explore changes to dietary habits and physical activity changes during the COVID-19 pandemic in Saudi Arabia. This questionnaire was comprised of four main sections with 27 questions in total and was developed and distributed in Arabic (See Additional file 1 for the English version). Ten experts in the nutrition field reviewed the initial questionnaire and were given a week to submit their comments. Based on their feedback, specific modifications were made and amended, such as correcting linguistic errors, rewording some questions, and adding questions to describe changes in the participants’ nutritional intake. The survey needed around 5–10 min to complete and was made available online for 2 weeks between June and early July 2020. Participants were informed at the beginning of the questionnaire about the study objective, the confidentiality of collected data, and the estimated time of completion.

The four sections of the questionnaire focused on personal and demographic details, anthropometric measurements, dietary habits and nutritional intake, and physical activity. The first section had eight questions about socio-demographic characteristics, including age, sex, nationality, place of residence, marital status, educational level, monthly house-hold income, and work status.

The second section assessed the participants’ anthropometric measurements, including self-reported weight in kilograms and height in centimeters which were used to calculate the body mass index (BMI). Participants were asked about their weight status—whether they had noticed any weight change during quarantine and to estimate the amount of weight gained or lost in kilograms.

The third section evaluated changes in the participants’ dietary habits and nutritional intake during quarantine. Participants were asked about the quantities of food they consumed as well as their frequency of snacking, consuming home-cooked food, consuming food from restaurants, and consuming healthy food. In the same section, they were asked about the possible reasons for their change in dietary habits during quarantine. Questions about whether they consumed immune-boosting food items or dietary supplements during quarantine were also included. Participants were also asked about their consumption of the following food items: fruits and vegetables, dairy products, meat, fish, poultry, sweets (cake, chocolate, and ice cream), savory snacks (chips and salty biscuits), sweetened juices and soft drinks, and drinking water. Regarding the food items, participants were asked to choose one of the following choices: increased intake, decreased intake, or no change in the intake.

In the fourth section, participants were asked about changes in their level of physical activity, frequency of physical activity per week, duration of physical activity per day, and types of physical activity performed during quarantine.

### Statistical analysis

A statistical analysis was performed using Minitab® statistical software (Version 19). The Anderson–Darling test was used to evaluate variables distribution. Categorical data were expressed as a number and a percentage; continuous data were expressed as mean and standard deviation. Differences between categorical variables were assessed with Chi-square test. A *P*-value of < 0.05 was statistically significant.

## Results

### Characteristics of the study participants

The survey was completed by 2255 participants. Table 1 presents the general characteristics of the studied population. Most participants were aged 30–39 years old (24%), female (64%), Saudi descent (91%), married (73%), and from the Western Region of Saudi Arabia (70%). Although most of the surveyed individuals had a received university-level education (68%), they showed a high percentage of unemployment (29%), and the majority had monthly incomes (34%) of 11,000–20,000 Saudi riyals.

**Table 1:** General characteristics of the study participants (n 2255)^a^

Variables	N	%
**Age (years)**		
18–29	484	21
30–39	534	24
40–49	447	20
50–59	469	21
≥ 60	321	14
**Sex**		
Male	802	36
Female	1453	64
**Nationality**		
Saudi	2058	91
Non-Saudi	197	9
**Marital Status**		
Single	446	20
Married	1653	73
Divorced	122	5
Widower	34	2
**Region**		
Western Region	1574	70
Central Region	357	16
Eastern Region	190	8
Northern Region	22	1
Southern Region	112	5
**Work Status**		
Student	229	10
Working remotely (from home)	541	24
Working at workplace	401	18
Retired	425	19
Unemployed	659	29
**Education Level**		
High school education or less	341	15
University education	1534	68
Higher education	380	17
**Income (Saudi riyals)**		
< 5000	236	10
5000–10,000	569	25
11,000–20,000	758	34
> 20,000	692	31

^a^Data presented as number and percentage

### Changes in body weight during the COVID-19 quarantine

Table 2 presents the participants’ BMI and weight changes during quarantine. According to the BMI category, most participants were overweight (36%), followed by obese and normal weight (31%). Of those surveyed, 38 and 26% reported an increase and decrease of weight, respectively, while 36% reported no change. Additionally, 34% reported a 0.0–0.9 kg weight change during quarantine.

**Table 2:** Participants’ BMI and weight changes during the COVID-19 quarantine (n 2255)^a^

Variables	Mean	SD
**Weight (kg)**	75.6	18.7
**BMI Category**^b^	**N**	**%**
Underweight	54	2
Normal weight	691	31
Overweight	803	36
Obese	707	31
**Weight Change**	**N**	**%**
Weight gain	859	38
Weight loss	582	26
No change	814	36
**Expected Weight Gained or Lost (kg)**	**N**	**%**
0.0–0.9	773	34
1.0–2.9	594	27
3.0–5.0	638	28
> 5.0	250	11

*SD* standard deviation, *BMI* body mass index

^a^Data presented as number and percentage unless otherwise stated

^b^Self-reported weight and height used to calculate the BMI. The BMI categories are underweight (< 18.5 kg/m2), normal weight (18.5–24.9 kg/m2), overweight (25.0–29.9 kg/m2), and obese (≥30 kg/m2)

### Changes in dietary habits during the COVID-19 quarantine

Table 3 summarizes the quantity of food consumption and frequency of snacking and eating home-cooked, restaurant, or healthy meals in different weight change groups. During quarantine, 40% of the surveyed participants consumed more food quantities and 45% snacked between meals more frequently. Increased amount of food consumption (29%) and the frequency of snacking (28%) were higher in individuals who gained weight compared to those who reported either losing weight or no weight change (*p* < 0.001). The majority of the participants reported incresed consumption of home-cooked (73%) and healthy meals (47%) during quarantine and dicreased consumption of foods from restaurants (7%). Consuming more home-cooked meals during quarantine was significantly associated with increased weight gain (*p* < 0.001). A significant difference was seen between the frequency of restaurant food consumption and weight changes during quarantine (*p* < 0.001). During the quarantine, increased consumption of healthy foods was significantly higher in individuals who lost weight compared to those who gained weight or those with no weight change (*p* < 0.001).

**Table 3:** Changes in dietary habits compared with weight changes during the COVID-19 quarantine in Saudi Arabia (n 2255)^a^

	Total (***n =*** 2255)	Weight Change			***P*** Value^b^
		Weight Gain (***n*** = 859)	Weight Loss (***n*** = 582)	No Change (***n*** = 814)	
**Quantity of Consumed Food**					< 0.001
Increased	894 (40)	657 (29)	62 (3)	175 (8)	
Decreased	483 (21)	16 (1)	371 (16)	96 (4)	
No change	878 (39)	186 (8)	149 (7)	543 (24)	
**Frequency of Snacking**					< 0.001
Increased	1022 (45)	641 (28)	132 (6)	249 (11)	
Decreased	424 (19)	36 (2)	289 (13)	99 (4)	
No change	809 (36)	182 (8)	161 (7)	466 (21)	
**Frequency of Consuming Home-Cooked Food**					< 0.001
Increased	1637 (73)	679 (30)	430 (19)	528 (24)	
Decreased	87 (4)	23 (1)	40 (2)	24 (1)	
No change	531 (23)	157 (7)	112 (5)	262 (11)	
**Frequency of Consuming Food from Restaurants**					< 0.001
Increased	154 (7)	82 (4)	21 (1)	51 (2)	
Decreased	1802 (80)	683 (30)	499 (22)	620 (28)	
No change	299 (13)	94 (4)	62 (3)	143 (6)	
**Frequency of Consuming Healthy Food**					< 0.001
Increased	1066 (47)	300 (13)	390 (18)	376 (16)	
Decreased	282 (13)	203 (9)	28 (1)	51 (3)	
No change	907 (40)	356 (16)	164 (7)	387 (17)	

^a^Data presented as number and percentage

^b^Differences between the three groups were assessed via Chi-square test

**Fig. 1:**
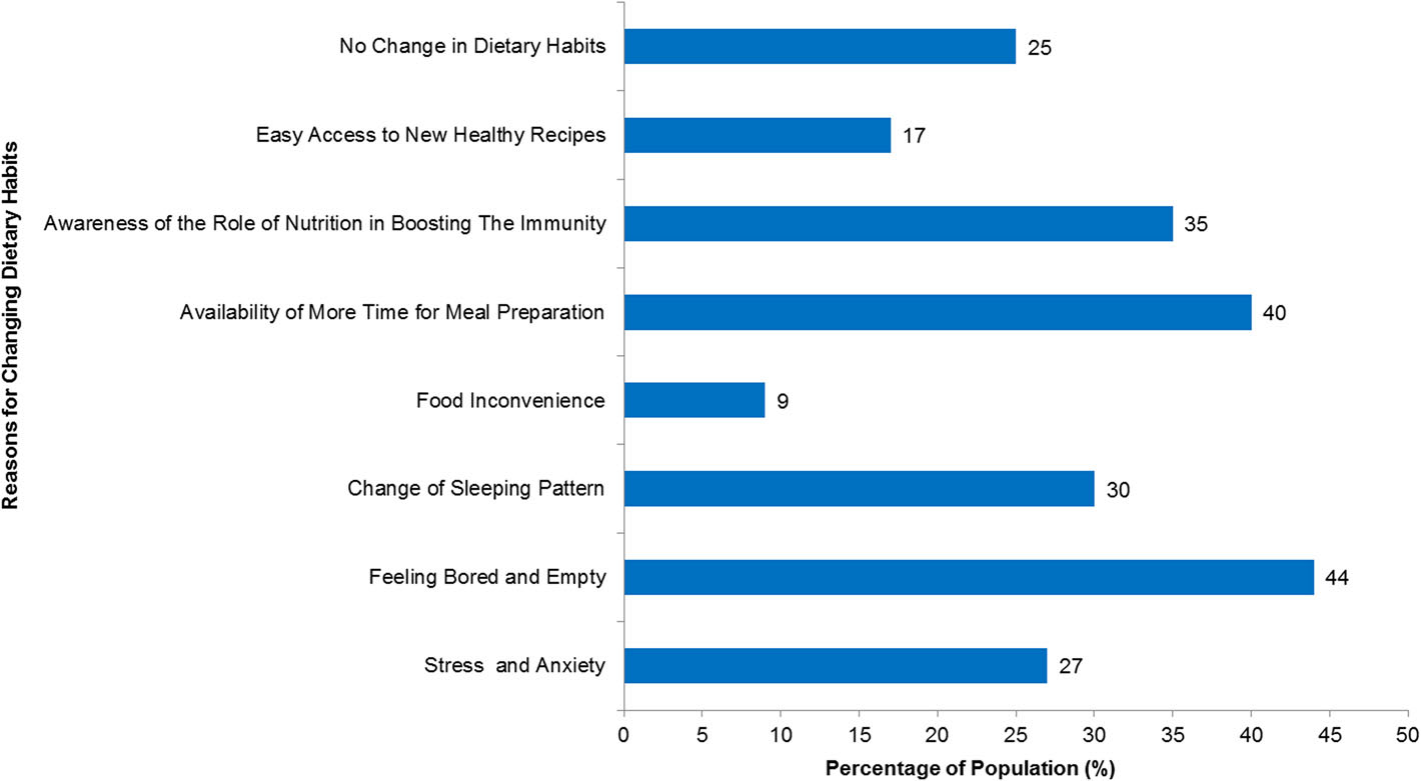
Reported reasons behind dietary habits changes during the COVID-19 quarantine (n 2255)

Fig. 1 shows some of the reasons behind the changing of dietary habits during quarantine. These changes have been attributed to weight change as a result of social and psychological factors, in addition to lower physical activity and changes in food consumption, as stated before. Feelings of boredom and emptiness (*n* = 991, 44%) and the availability of time to prepare meals (*n* = 905, 40%) were the most reported reasons. The surveyed individuals also reported easy access to new recipes, stress and anxiety, changing sleep patterns, and knowing more about the role of nutrition in boosting immunity as other explanations for changing their dietary habits. Only 9% (*n* = 212) of participants stated that their quarantine dietary behavior resulted from the inconvenience of food.

### Changes in nutritional intake during the COVID-19 quarantine

**Fig. 2:**
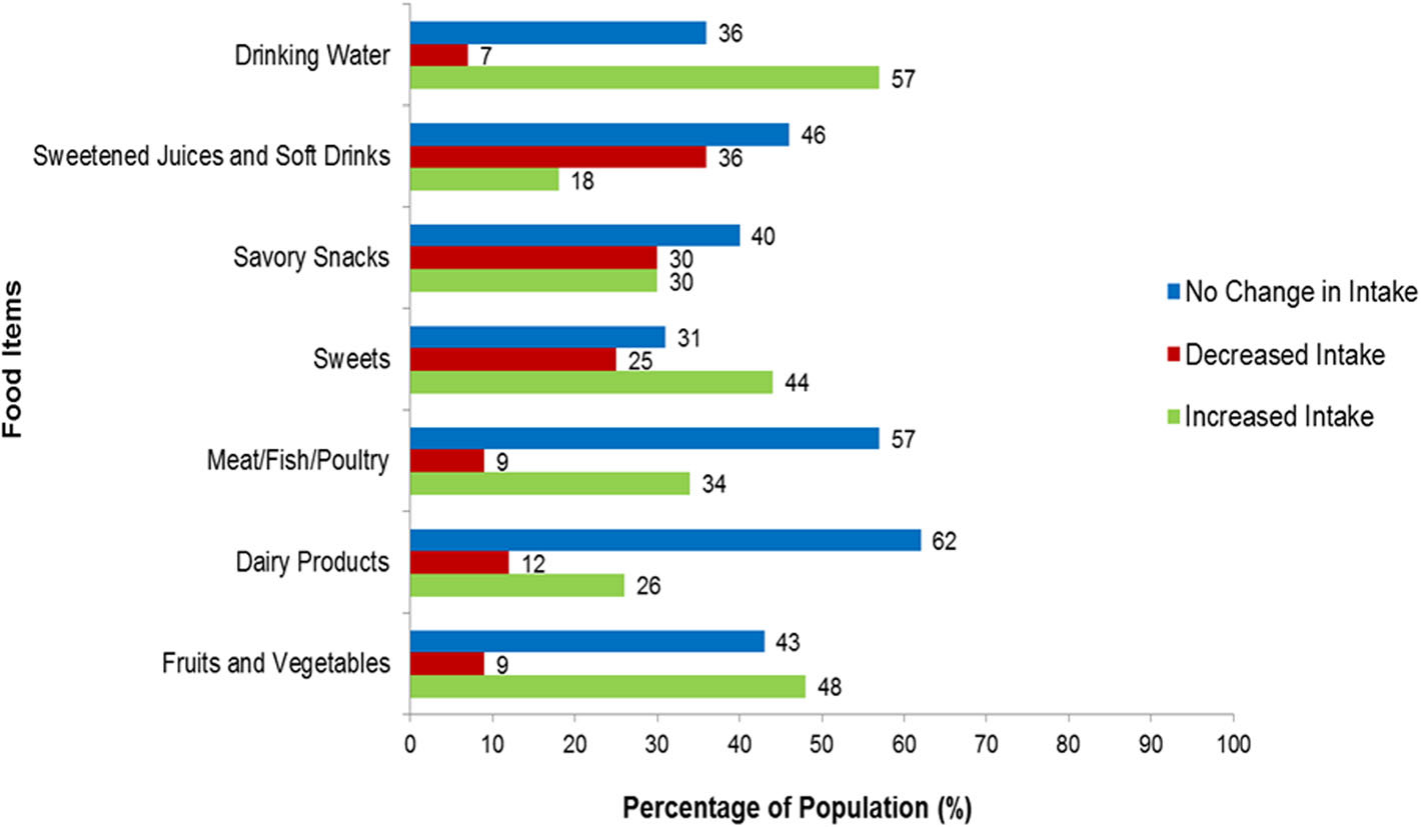
Changes in the consumption of particular food items during the COVID-19 quarantine period compared with the usual intake (n 2255)

The present study also compared changes in the consumption of particular food items during quarantine in relation to the usual intake (Fig. 2). While 57% of participants admitted no changes in their consumption of meats, they did increase their intake of drinking water. There were also similar proportions of surveyed individuals who either increased (48%) or did not change (43%) their intake of fruits and vegetables during quarantine. Although 44% of subjects increased their sweets intake, 46% did not show any changes in their consumption of sweetened juices and soft drinks.

### Immune-boosting foods and dietary supplements

Half of the participants (*n* = 1141, 50%) reported eating immune-boosting foods and 34% (*n* = 772) reported consuming dietary supplements during quarantine. In regards to immune-boosting foods, honey (43%), lemon (40%), and ginger (32%), orange (18%), black seed (17%), turmeric (16%), fresh fruits and vegetables (13%) and garlic (10%) being the most consumed. Vitamin C was the most consumed dietary supplement during quarantine (*n* = 386, 50%), while 22% (*n* = 169) and 26% (*n* = 198) of participants indicated taking vitamin D and multivitamins, respectively. Other dietary supplements reported include omega-3 (8%), B vitamins (7%), iron (6%), zinc (6%), calcium (4%) and magnesium (2%).

### Changes in physical activity level during the COVID-19 quarantine

**Fig. 3:**
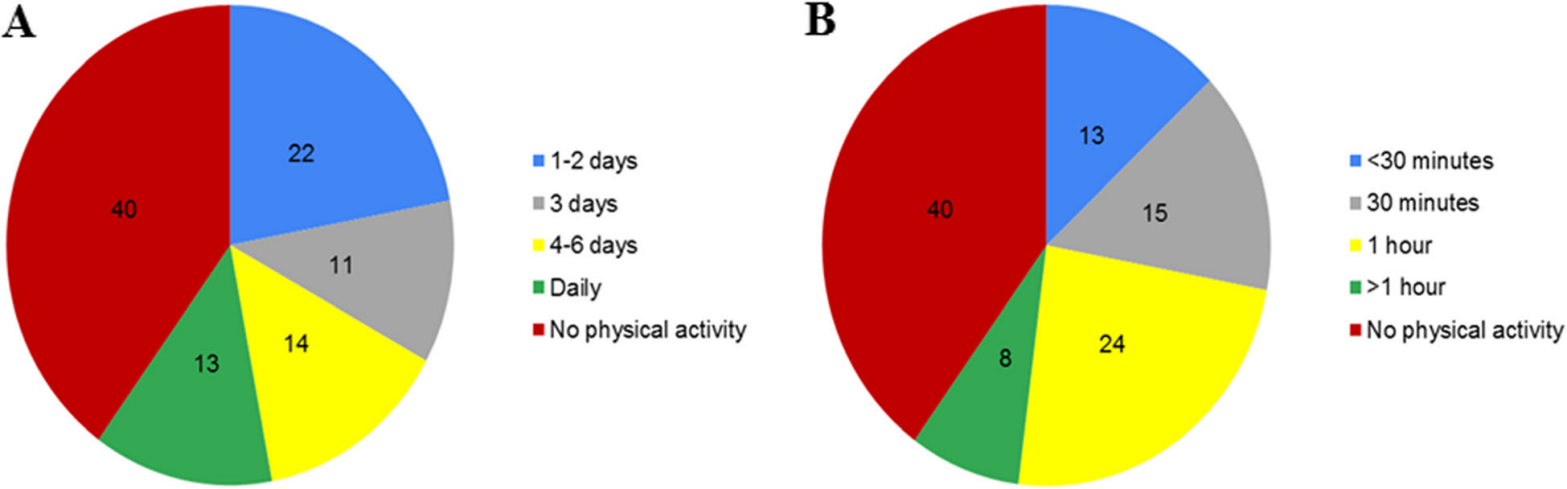
Frequency of physical activity per week (a) and duration per day (b) during the COVID-19 quarantine (n 2255)

Table 4 reports changes in the participants’ physical activity during quarantine, which decreased in 1181 (52%) subjects, increased in 603 (27%), and did not change in 471 (21%). Figure 3 presents the frequency and duration of the participants’ physical activity during quarantine. Out of 2255 participants, 902 (40%) did not perform any physical activity; 496 (22%) performed physical activity 1–2 days per week (Fig. 3a). The majority of respondents who exercised (24%) reported having one hour of physical activity per day during quarantine (Fig. 3b). Thus, decreased physical activity levels was significantly higher in individuals who reported weight gain (*n* = 624, 28%) compared to those who lost weight or those with no weight change during quarantine (*p* < 0.001) (Table 4).

**Table 4:** Changes in the level of physical activity compared to weight changes during the COVID-19 quarantine in Saudi Arabia (n 2255)^a^

	Total (***n =*** 2255)	Weight change			
		Weight Gain (***n =*** 859)	Weight Loss (***n =*** 582)	No Change (***n =*** 814)	***P*** value^b^
**Level of Physical Activity**					< 0.001
Increased	603 (27)	122 (5)	286 (13)	195 (9)	
Decreased	1181 (52)	624 (28)	217 (9)	340 (15)	
No change	471 (21)	113 (5)	79 (4)	279 (12)	

^a^Data presented as number and percentage

^b^Differences between the three groups were assessed via Chi-square test

There were different types of physical activities declared by the individuals who performed physical activity during the lockdown (*n* = 1415). The most common physical activity performed during quarantine was walking (65%). Other physical activities the participants reported include cardio (11%) and resistance (7%) exercises. Minimal practices of home exercises such as yoga, home training, dancing, zumba, running, and cycling were also reported.

## Discussion

To the best of our knowledge, this is the first study to evaluate the influence of COVID-19-related home confinement on changes in weight, dietary habits and physical activity levels in a large sample of Saudi Arabian adult population. Overall, this study has found that quarantine has negatively affected body weight, dietary habits, and physical activity levels.

Data from the current study showed that the majority of the participants (38%) reported weight gain during the lockdown, with a reported gain of around 3–5 kg. Similarly, other studies have reported weight gain of ~ 3–4.5 kg during quarantine [10]. Since quarantine is associated with limiting people’s ability to go to work, the gym, parks, and even practicing normal daily routines, weight gain is expected due to the general decrease in energy expenditure. Moreover, the emotional distress accompanied with being locked at home for months and fear of the novelty and the high spread of COVID-19 [18] might provoke emotional eating and cravings. This result is in line with those of recent studies that captured weight gain concerning COVID-19 home confinement [6, 10, 19]. In this study, however, a comparable percentage of participants (36%) did not notice any weight change. This could be due to an increased level of awareness, or they may have not been as majorly affected by quarantine as people who continued to go to their workplaces during the curfew in comparison to the weight gain group.

The link between the current global lockdown with higher amounts of food intake was previously reported in Poland, Italy and UK [10, 11, 14] . Consistent with previous studies, the present study has demonstrated an increase in food consumption and snacking which were significantly higher in those who gained weight during quarantine. A logical explanation is the nature of quarantine, with people spending most of the day locked at homes with minimal activities available, watching more television and having an abundance of stocked groceries. Prior evidence has shown that increased time spent on watching television was associated with increased risk of weight gain in adults [20]. The availability of large food quantities for many days might lead to overeating, not necessarily due to hunger [21]. During home confinement periods, people tended to stock their kitchens with different foods to reduce unnecessary grocery trips due to the fear of contracting the infection [10]. The majority of these foods are ready-to-eat meals, canned foods, and products with long-shelf-life, which are often dense with calories.

Cooking at home is usually perceived as healthier or at least lower in calories than eating from restaurants. Surprisingly, almost 30% of participants in this study who reported an increase in their home-cooked meals than before the lockdown had gained weight during the quarantine. This result might be elaborated by the fact that not all cooking methods are considered healthy; some people might add large amounts of fat and/or sugar, which could lead to a substantial amount of added calories. Although such ingredients are high in calories, they play a role in increasing the palatability of food, making it more appealing in such stressful situations. Interestingly, it was found by Rodríguez-Pérez et al. [12] that people in Spain have increased their Google searches of the term “homemade cake” since the start of the lockdown. Homemade bread and cakes intake were also found to be higher during quarantine in Italy [11, 19]; therefore, a similar trend in food and cooking could possibly be applied in Saudi Arabia. However, 19% of those who reported an increase in home-cooked meals have lost weight, which is apparently due to a healthier and less caloric way of cooking. This finding is consistent with that of Rodriguez-Perez et al. [12] who found a better adherence to healthy types of cooking during the COVID-19 quarantine among the Spanish population. The quarantine is likely to have wide ranging effects on population’s dietary habits. Despite all the negative dietary changes, it was found previously that staying at home during the quarantine period was an opportunity for adapting positive dietary behaviors [22]. The current study supports this finding and showed that around half of the study participants increased their intake of healthy food; however, the definition “healthy food” was not stated in the question and was thus dependent on the perception of each individual. A significantly higher percentage of those who reported an increase in healthy food consumption expressed a decrease in body weight compared to other groups.

The main two reasons indicated in the study sample for changing dietary habits during the quarantine were more due to boredom and emptiness or having more time for meals preparation. Staying home for long periods may raise the feeling of boredom, which is often associated with overeating to escape monotony [23]. This behavior was also reported by Zachary et al. [6] where participants stated an increase in eating with others, eating because of cravings, and eating in response to stress and boredom.

The current study also inspected changes in the intake of particular foods during the lockdown. Higher water intake was reported by more than half of the participants. This is a positive behavior for the Saudi population, as hydration status has been linked to innate mucosal immunity [24]. This behavior might because of easier access to water and increased awareness of the amount of water consumption during quarantine. An Italian study also showed a sufficient water drinking habit among the Italian population [19]. Similar to the findings of other studies [10, 11, 19], the majority of subjects in the present study have shown an increase in the intake of sweets, including cakes, chocolate, and ice cream. This also accords with this study’s observation of dietary patterns, which showed that subjects in this study increased their snacking frequency during quarantine; thus, it can be assumed that the bulk of snacks consumed were sugary ones rather than savory. During confinement, people were facing incredibly stressful conditions, between continuously reading or watching updates in the news and being afraid to get infected with the COVID-19. Subsequently, stress can urge people to have food cravings, especially to sweets known as “comfort food,” which are loaded with calories [25]. Craving for carbohydrates, in particular, stimulates the production of serotonin (a neurotransmitter found in the brain), which positively affects mood [26]. Such behavior, in turn, could make people at a higher risk for obesity and serious COVID-19 complications. The results of this study also showed that almost half of the subjects increased their fruit and vegetable consumption. This is noteworthy, particularly in a society that relies on an omnivorous diet that is rich in red meat and poor in fruits and vegetables, as discussed by Afshin et al. [27]. Regarding the intake of fruits and vegetables during quarantine in other studies, findings were inconsistent. The intake of fruits and vegetables were increased in Spain [12], decreased in Poland [10], and did not changed in Italy [19].

The study revealed that the most commonly consumed natural food during quarantine was honey, followed by lemon. Consuming honey is part of Saudi culture; furthermore, honey is known for its general potent antiviral effects by many researchers [28–30]. Some efforts have been conducted in Saudi Arabia to examine the effect of honey and other natural products on COVID-19 patients [31]. In regards to dietary supplements, this study revealed that half of the participants who admitted to taking dietary supplements reported that they were taking vitamin C supplements, while a quarter reported the intake of multivitamins and vitamin D. It is unsurprising that vitamin C was the most consumed dietary supplement, as it is well known for its immune-boosting effects, especially in individuals with subnormal levels of the vitamin, as stated by Carr et al. [32].

The Saudi population is at a low physical activity level compared to other populations [33], which was exacerbated by the lockdown with a decline that was found in this study. Around half of the population reported not practicing any kind of physical activity during the quarantine period; however, the other half reported performing 60–120 min per week of moderate intensity exercise (predominantly walking indoors or outdoors), which is still less than the recommended level of physical activity to provide protective effects against chronic diseases [34]. This study supports recent evidence from an international observation showing a universal decline in all physical activity levels during COVID-19 pandemic [35].

The study was done in a relatively short period of time as suggested by previous studies [36]. It also took place amid the pandemic’s highest restrictions imposed in Saudi Arabia, that is part of the Middle East and shares many cultural, habitual, and dietary behaviors that can also provide insights into neighboring countries. To the best of the authors’ knowledge, this is the first study to provide the previously mentioned insights in Saudi Arabia. Although this study might be specific to certain circumstances, the outcomes and results are significant in the prevention and preparation of any future incidents that necessitates a lockdown.

Although this study provides a general insight on how dietary habits and physical activity changed during the pandemic, it has some limitations. Many considerations were put in mind when structuring the questionnaire to encourage all societal groups to participate. Although the questionnaire was relatively short and used simple language, it was limited in providing specific information, such as the exact quantities of food consumed and details about food preparation methods. It is also evident that this study used self-reported information, including weight and height measurements and expected weight changes during quarantine due to its anonymous nature, and thus might introduce misreported data. Although data were collected from all regions of Saudi Arabia, the variations in dietary habits and lifestyle between these regions were not considered. However, the aim of this study focused on the overall dietary habits and physical activity changes in the whole country.

## Conclusion

The present study is the first to provide data regarding dietary habits and physical activity during the COVID-19 home confinement period in Saudi Arabia. The majority of the population have shown weight gain, increased food consumption, and decreased physical activity. In particular, subjects who gained weight tended to consume more snacks, larger food quantities, and showed lower physical activity levels. A trend toward healthier food intake compared to regular diets was seen, including increased intake of fruits, vegetables, and water and decreased intake of sweetened juices and soft drinks. The intake of meats and sweets, meanwhile, either increased or did not modify in most subjects. Assessing the changes in populations’ dietary habits during the lockdown may help with understanding the implications surrounding the population’s health and wellbeing later in life. For that reason, further larger-scale studies should be undertaken to investigate if the COVID-19 lockdown would result in a persistent effect on dietary habits and physical activity.

## Supplementary Information

**Additional file 1.** English version of the questionnaire.

## Data Availability

All data generated or analysed during this study are included in this published article.

## Abbreviations

WHO: World health organization
BMI: Body mass index
