# Supplementary material for: The impact of COVID-19 quarantine on dietary habits and physical activity in Saudi Arabia: a cross-sectional study
Source: BMC Public Health. 2021 Jul 30;21:1487. doi: 10.1186/s12889-021-11540-y (PMC8323088; doi:10.1186/s12889-021-11540-y)
Supplement: Supplementary file 1 — Additional file 1. English version of the questionnaire. [file 12889_2021_11540_MOESM1_ESM.pdf]

Title: The impact of COVID-19 quarantine on dietary habits and physical activity in Saudi Arabia: a cross-sectional study

Additional file 1

1) **Sex**
   Male
   Female

2) **Age**
   18-29
   30-39
   40-49
   50-59
   60-64
   65 and more

3) **Nationality**
   Saudi
   Non-Saudi

4) **Marital status**
   Single
   Married
   Divorced
   Widower

5) **Region**
   Eastern
   Riyadh
   Alqaseem
   Najran
   Aseer
   Jazan
   Albaha
   Makkah Almukarrama
   Almadena Almunawara
   Hail
   Tabouk
   Aljouf
   Northern boarders
   Mention the name of your city ………..

6) **Work status during the Corona pandemic**
   Working remotely (from home)
   Working at workplace
   Student
   Retired
   Unemployed

7) **Education**
Primary education
Intermediate education
High school education
Diploma
University education
Higher education

8) **Income (Saudi riyals)**
Less than 5000
5000-10,000
11,000-20,000
More than 20,000

9) **What is your current weight in (kg)?** …………

10) **What is your height in (cm**)? …………

11) **Have you noticed any changes in your weight during the COVID-19 quarantine period?**
Weight gain
Weight loss
No change in weight

12) **In case of weight gain or loss, how many kilograms are expected?**
0-0.9 kg
1-2.9 kg
3-5 kg
More than 5 kg

13) **Have you changed the quantity of food consumed during the COVID-19 quarantine period compared to the amount usually consumed?**
Yes, increased
Yes, reduced
No change in the quantity of food consumed

14) **Have you changed the frequency of snacking between the main meals during the COVID-19 quarantine period compared to the number of snacks usually consumed?**
Yes, increased
Yes, reduced
No change in the frequency of snacking

15) **Have you changed the intake of home-cooked food during the COVID-19 quarantine period compared to your usual intake?**
Yes, increased
Yes, reduced
No change in the intake of home-cooked food

16) **Have you changed the intake of food from restaurants during the COVID-19 quarantine period compared to the usual intake?**
Yes, increased
Yes, reduced
No change in the intake of food from restaurants

17) **Have you changed the intake of healthy food during the COVID-19 quarantine period compared to the usual intake?**
Increased
Reduced
No change in the intake of healthy food

18) **Have you changed the amount of intake of the following food items during the COVID-19 quarantine period compared to the amount usually consumed?**

Food Item	Yes, increased	Yes, reduced	No, did not change
**Fruits and Vegetables**			
**Dairy products**			
**Meat, chicken, fish**			
**Sweets (cake, chocolate, ice cream, biscuit)**			
**Savoury (chips, biscuit)**			
**Soft drinks, sweetened juices**			
**Drinking Water**			

19) **Did you eat any natural food items to boost your immunity level during the COVID-19 quarantine period?**
Yes
No

20) **If yes, please specify**………

21) **Did you take any nutritional supplements such as vitamins or minerals to boost your immunity during the COVID-19 quarantine period?**
Yes
No

22) **If yes, please specify**........

23) **What are the reasons for changing your dietary habits during the COVID-19 quarantine period? (More than one answer can be selected)**
Stress and anxiety
Feeling bored and empty
Change of sleeping pattern
Availability of more time for meals preparation
Increased awareness of the role of nutrition on boosting the immunity
Food inconvenience
Easy access to new healthy recipes
No change in dietary habits

24) Have you changed your daily physical activity level during the COVID-19 quarantine period compared to your usual physical activity level?

Yes, increased
Yes, reduced
No change in physical activity level

25) How many times a week have you performed physical activity during the COVID-19 quarantine period?

1-2 days per week
3 days per week
4-6 days per week
Daily
No physical activity

26) How many hours a day have you allocated to physical activity during the COVID-19 quarantine period?

Less than 30 minutes
30 minutes
30 minutes - 1 hour
More than one hour
No physical activity

27) Please specify the type of physical activity you performed during the COVID-19 quarantine period?

Thank you for your participation and support
